# Cystoscopic retrieval of a migrated IUD with bladder stone formation during pregnancy termination: a Case Report

**DOI:** 10.3389/fmed.2025.1731963

**Published:** 2026-01-12

**Authors:** Yanhua Zhang, Jianbo Zhou, Wenlei Yao, Lingyan Zhang, Haifeng Gao, Feng Han, Feng Lu

**Affiliations:** Binhai County People’s Hospital, Yancheng, China

**Keywords:** intrauterine device, uterine perforation, bladder calculus, cesarean section, case report

## Abstract

**Background:**

Uterine perforation with intravesical migration is an exceedingly rare complication of intrauterine device (IUD) use. Its non-specific symptomatology often leads to delayed diagnosis, while the presence of a uterine scar, such as from a prior cesarean section, may significantly increase the risk.

**Case presentation:**

A 38-years-old woman (gravida 5, para 2) with a history of two cesarean sections presented at 8 weeks of gestation for termination of pregnancy. A V-shaped copper IUD had been placed over 10 years earlier. Notably, she reported a 1-year history of intermittent urinary frequency and gross hematuria, which were partially relieved by hydration. Pelvic computed tomography (CT) demonstrated that the IUD had perforated the lower anterior uterine wall at the site of the previous cesarean scar, with one arm penetrating into the bladder lumen and becoming encased by a 5 mm × 3 mm × 2 mm calculus.

**Intervention and outcome:**

Under general anesthesia, the patient first underwent an uncomplicated hysteroscopic abortion. This was followed by cystoscopy, which confirmed the intravesical IUD arm embedded in the stone. The entire complex was successfully removed intact via transurethral extraction using grasping forceps, without the need for lithotripsy. A urethral catheter was left indwelling postoperatively for 4 weeks (protocol target 2–3 weeks; delayed at patient’s request) to facilitate bladder healing. Upon its removal, the patient’s voiding symptoms and hematuria had completely resolved. At 4-months follow-up, she remained asymptomatic.

**Conclusion:**

This case highlights that a prior cesarean section scar is a paramount risk factor for late-onset IUD migration into the bladder. Unexplained lower urinary tract symptoms in women with this history warrant prompt investigation with cross-sectional imaging such as CT. When the migrated IUD is associated with a small, manageable stone, direct cystoscopic retrieval is a viable and effective minimally invasive treatment option.

## Introduction

The intrauterine device (IUD) is widely used globally as a long-acting reversible contraceptive method due to its high efficacy, cost-effectiveness, and convenience (1). Despite its excellent safety profile, it is not without risks, which include contraceptive failure (intrauterine or ectopic pregnancy) and the less common complication of uterine perforation (2–4).

Intrauterine device migration is a serious but relatively rare complication, with an estimated incidence ranging from 1 to 2 per 1,000 cases (2, 5). Risk factors associated with IUD migration include insufficient operator experience, abnormal uterine position (such as extreme anteversion or retroversion), and inappropriate timing of insertion–defined as placement within 4 weeks after delivery, especially after vaginal delivery, or while breastfeeding–which significantly increases the risks of both complete expulsion (adjusted risk ratio 5.3–8.3) (6) and uterine perforation (incidence ≈ 1–2 per 1,000 insertions) (7). After migrating into the abdominal cavity, the displaced IUD may cause erosion to adjacent organs, such as the omentum, intestines, or bladder (5, 8, 9). Given that this process often follows a chronic and insidious course, patients may remain asymptomatic in the early stages until serious complications–including intestinal obstruction, bowel strangulation, or fistula formation–develop and bring the condition to clinical attention (8, 10). If the IUD migrates into the bladder and acts as a nidus for secondary vesical calculus formation, patients may present with refractory urinary irritation symptoms and hematuria (9, 11). Since empirical antibiotic therapy typically yields no significant improvement, the diagnosis is often established at this stage (11). Management of a displaced IUD requires comprehensive clinical evaluation, and surgical removal is generally indicated. Available approaches include hysteroscopy, cystoscopy, laparoscopy, or laparotomy, depending on the location and associated complications (12–14).

This report presents a case of a woman with two previous cesarean deliveries whose IUD migrated into the bladder and formed stones, which were ultimately successfully removed via cystoscopy without lithotripsy. This case aims to enhance clinical awareness of this rare entity, elucidate the pathophysiological link between cesarean scars and IUD migration, and discuss the role of individualized minimally invasive management strategies.

## Case report

### History and presentation

A 38-years-old woman, gravida 5, para 2 was admitted to our gynecology department at 8 weeks of gestation requesting termination of pregnancy. Her medical history was significant for two previous lower-segment cesarean sections. A V-shaped copper IUD had been inserted over 10 years prior. Notably, for approximately 1 year before admission, she had experienced intermittent urinary frequency and passage of pinkish urine. She described the hematuria as occasional, non-cyclical, and precipitated by exercise; she denied any urinary incontinence. CT cystography was offered but declined because of radiation anxiety. Pre-operative counseling specifically included the possibility of a large vesico-uterine fistula, potential intra-operative urology consultation, conversion to laparotomy and layered bladder repair. These symptoms partially alleviated with increased fluid intake, leading her to dismiss their significance and not seek medical attention.

### Diagnostic work-up

**FIGURE 1:**
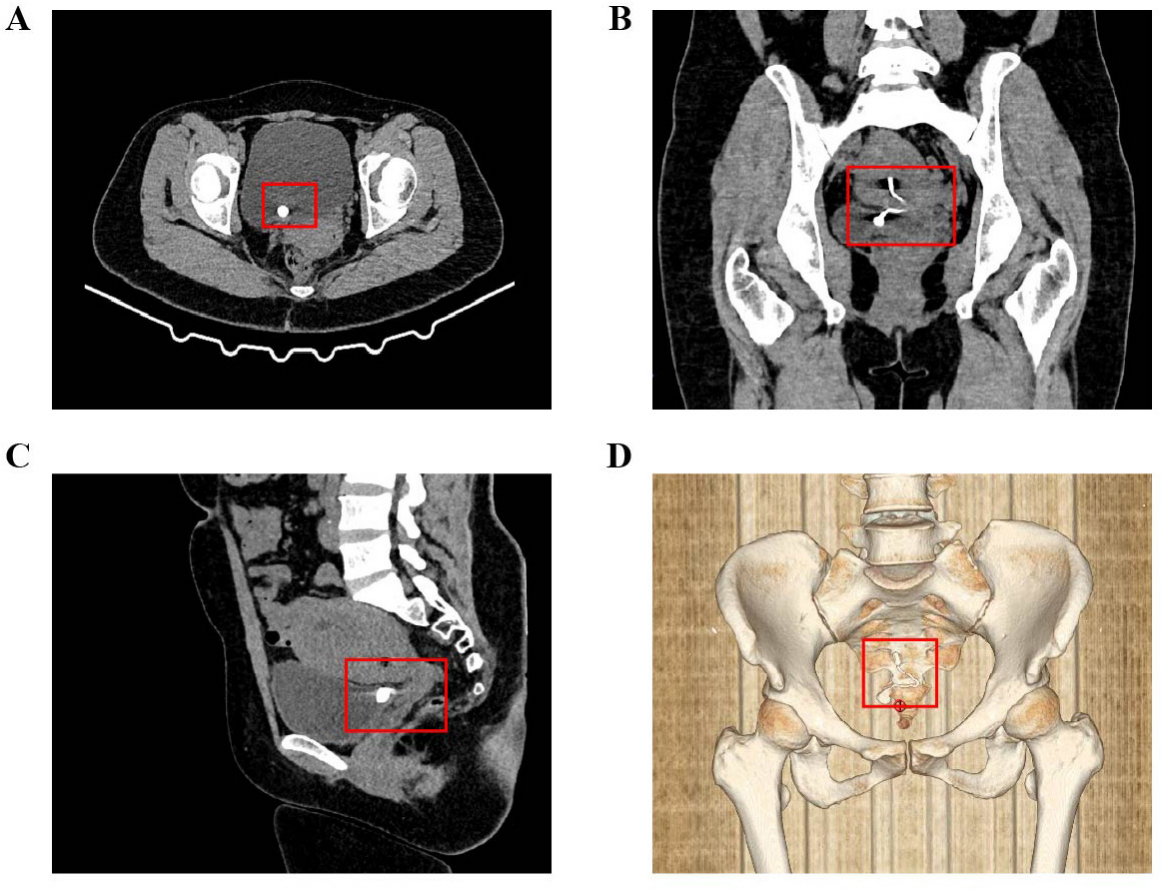
Preoperative pelvic CT imaging. **(A)** Axial view showing the IUD penetrating the anterior uterine wall into the bladder lumen, encased by a vesical calculus. **(B)** Coronal view of the complete V-shaped IUD with marked thickening of the intravesical portion due to stone adherence. **(C)** Sagittal view illustrating the point of exit from the lower anterior uterine wall near the cervix. **(D)** Three-dimensional CT reconstruction of the intact V-shaped IUD, highlighting the thickened bladder-embedded segment.

An initial transvaginal ultrasound confirmed an intrauterine viable fetus. Due to an empty urinary bladder during the examination, the IUD was observed partially embedded within the myometrium, and a calculus was noted at the distal ureter. Suspecting IUD embedment, the patient was hospitalized for further evaluation. To accurately determine the precise location of the IUD, pelvic computed tomography (CT) was performed. The CT scan clearly revealed that the V-shaped IUD had perforated through the anterior wall of the lower uterine segment, precisely at the site of the previous cesarean scar. One arm of the IUD was completely embedded within the bladder lumen and encased by a 5 mm × 3 mm × 2 mm calculus (Figures 1A–D).

### Treatment and outcomes

**FIGURE 2:**
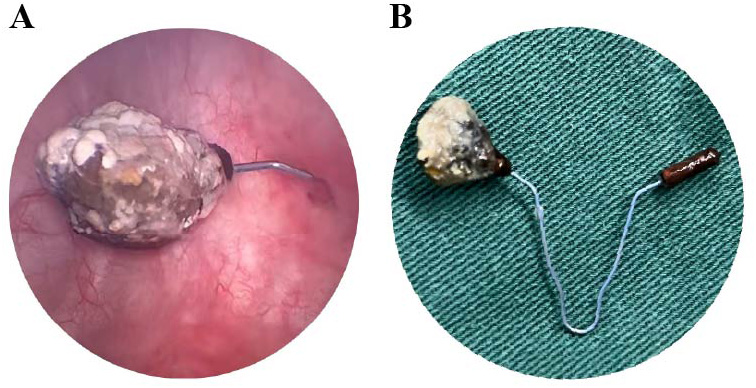
Intraoperative cystoscopic views and retrieved specimen. **(A)** Cystoscopic image showing one arm of the IUD penetrating the bladder wall, with surface stone encasement. **(B)** The intact V-shaped IUD after transurethral extraction, with the complete vesical calculus adherent to its surface.

A multidisciplinary team of gynecologists and urologists developed and executed a combined surgical plan. Under general anesthesia, the patient first underwent hysteroscopy and suction curettage to manage the abortion. During hysteroscopy, a small metallic fragment of the IUD was observed protruding through the cesarean scar niche, though no image was captured. Subsequent cystoscopy revealed one arm of the V-shaped IUD encased within a firm calculus measuring 5 mm × 3 mm × 2 mm (Figure 2A). As the calculus was adherent but not bulky, lithotripsy was deemed unnecessary. The entire IUD, along with the encapsulating calculus, was successfully removed intact transurethrally using grasping forceps (Figure 2B). After removal, cystoscopic evaluation of the original perforation site showed only a 2-mm mucosal punctum. This lesion was managed conservatively without electrocautery or suture. A urethral catheter was placed postoperatively to facilitate bladder healing and was retained for 4 weeks–longer than the protocol target of 2–3 weeks, per the patient’s request. The patient recovered well without complications. At the 4-months follow-up (including both face-to-face and telephone assessments), she remained asymptomatic, with normal urinalysis and pelvic ultrasound findings. Follow-up is planned at 6 and 12 months, followed by annual telephone consultations for 2 years to monitor for potential late-onset vesico-uterine fistula. Prior to discharge, she was advised to use male condoms for temporary contraception; a definitive method will be discussed after completion of urological follow-up.

## Discussion

This case report exemplifies a rare and clinically significant complication of long-term IUD use: chronic transmural migration culminating in bladder perforation and secondary stone formation. The principal findings encompass the intravesical migration of a copper IUD, the formation of a sizable calculus encasing its intravesical arm, and the successful resolution through a multidisciplinary team MDT, minimally invasive surgical approach.

A paramount risk factor identified in our patient was her history of two previous cesarean sections. The resultant scar in the lower uterine segment, a recognized locus of structural weakness often termed a “cesarean scar niche,” likely offered diminished resistance to penetration and guided the IUD’s trajectory anteriorly toward the bladder (15). Recent quantitative data underscore this risk: in a pooled analysis of 7,661 postpartum IUD insertions the complete expulsion rate was 3.8% after cesarean delivery but 14.8% after vaginal delivery (adjusted RR 4.57; 95% CI 3.49–5.99) (6), while the APEX-IUD cohort documented a doubling of uterine perforation risk when insertion occurred within 36 weeks of cesarean birth (7). This indolent nature of migration directly led to a marked diagnostic delay, as the patient’s non-specific urinary symptoms were initially misleadingly attributed to common causes, a phenomenon frequently reported in the literature (12, 16–18). This case underscores a vital clinical imperative: in any woman with a long-term IUD, particularly with a prior cesarean section, new or persistent lower urinary tract symptoms must raise suspicion for IUD migration.

The pathogenesis of IUD-induced bladder stones is multifactorial. The displaced IUD initiates the process by serving as a nucleation scaffold for crystals, consistent with crystallization theories (19). Copper release then promotes mineralization via an inflammatory response, a recognized pathway in stone formation (20). The foreign body also facilitates urease-producing bacterial colonization, resulting in infection stones (21, 22). This comprehensive mechanism explains the inefficacy of empirical antibiotics and the persistence of symptoms (11, 23).

Furthermore, this case underscores the critical importance of precise preoperative imaging in preventing iatrogenic complications. Pelvic CT proved indispensable by clearly demonstrating complete uterine wall perforation by the IUD, with its intravesical segment encased within a rigid, hook-shaped calculus (Figures 1A–D). Attempted blind hysteroscopic retrieval based solely on ultrasound findings could have resulted in avulsion of this structure, potentially causing significant bladder laceration. This experience reinforces existing recommendations that for any missing IUD, confirming its extrauterine location and spatial relationships through cross-sectional imaging is essential for ensuring safe management (24, 25).

The definitive treatment strategy was guided by this detailed preoperative assessment. CT findings of a relatively small and adherent calculus permitted minimally invasive transurethral extraction under cystoscopic guidance without requiring lithotripsy. Management was further individualized based on the nature of the injury; given the full-thickness bladder wall perforation, prolonged postoperative urinary catheterization was implemented to promote spontaneous healing through continuous decompression, consistent with the principle of promoting healing by reducing tension on the wound (26). Although the cystoscopic defect was only 2 mm, we recognize that vesico-uterine fistulae can declare themselves months later; hence prolonged, structured follow-up was arranged. The successful outcome ultimately depended on seamless MDT collaboration between gynecology and urology services, spanning from joint preoperative planning to coordinated postoperative care.

## Conclusion

This case provides several critical contributions to clinical practice, despite the inherent limitations of a single case report that may affect generalizability. It emphasizes the necessity of maintaining a high index of suspicion for IUD migration in at-risk patients, particularly those with a history of uterine surgery, as asymptomatic or atypical presentations can delay diagnosis (10, 27). Furthermore, it underscores the indispensable role of cross-sectional imaging, such as CT, in precise preoperative planning to avoid catastrophic complications during retrieval (28, 29). Finally, it exemplifies how a meticulously planned, MDT approach–integrating gynecology and urology–forms the cornerstone of managing this complex condition, thereby ensuring patient safety and successful clinical outcomes.

## Data Availability

The original contributions presented in this study are included in this article/supplementary material, further inquiries can be directed to the corresponding author.
